# Rhabdomyolysis Following Prolonged Entrapment on a Fence: A Case Report

**DOI:** 10.7759/cureus.51954

**Published:** 2024-01-09

**Authors:** Chukwuemeka Nwaneri, Ahmed M Aboshehata, Adrian R Marsh

**Affiliations:** 1 Department of Emergency Medicine, Shrewsbury and Telford Hospital NHS Trust, Royal Shrewsbury Hospital, Shrewsbury, GBR

**Keywords:** rhabdomyolysis and compartment syndrome, entrapment, prolonged immobilization, alcohol, acute renal injury, rhabdomyolysis

## Abstract

It is well documented that prolonged immobilization and heavy alcohol consumption can independently cause rhabdomyolysis; however, entrapment on a fence following alcohol consumption resulting in rhabdomyolysis without prolonged coma or seizures has not been reported. We report a case of a 25-year-old man who, following alcohol consumption, whilst attempting to climb a fence, became entrapped and desperately had to clinch on the fence with both forearms for over three hours, resulting in rhabdomyolysis. This case report highlights the importance of recognizing the potential complications associated with prolonged immobilization and the subsequent management of rhabdomyolysis.

## Introduction

Rhabdomyolysis is a condition characterized by the breakdown of skeletal muscle fibers, with the release of intracellular contents into the bloodstream [1], with acute kidney injury (AKI) being the most critical complication. The diagnosis is confirmed as an increase in creatine kinase (CK) to five to tenfold the upper limit of normal and positive urine myoglobin [2,3]. Irrespective of the underlying cause, the muscle injury initiates a series of actions that release extracellular calcium ions into the cell's interior. These excess calcium ions lead to abnormal interactions of actin and myosin, which activate cellular proteases that cause destruction and necrosis of muscles. Large quantities of potassium, myoglobin, phosphate, creatine phosphokinase, and urate leak into the extracellular space and then into the circulation. In addition, muscle cell hypoxia depletes adenosine triphosphate (ATP) levels [4]. CK levels rise two to 12 hours after the onset of an assault of muscle injury and peak three to five days after injury. However, if continuing muscle injury is absent, serum myoglobin levels may return to normal within one to six hours [5]. When the kidney is exposed to high levels of myoglobin, it causes the distinctive kidney damage observed in cases of rhabdomyolysis, which can potentially develop into acute renal failure. A rare consequence of rhabdomyolysis is progression to acute compartment syndrome [6]. We present a case of rhabdomyolysis in a young male who experienced prolonged entrapment on a fence after drinking alcohol, resulting in significant muscle damage and subsequent release of myoglobin into the bloodstream.

## Case presentation

A 25-year-old man was brought to the emergency department by the police after finding him clinching on a fence close to his house. He was not under arrest. The patient stated that he had consumed a considerable amount of alcohol that night, over 10 pints of beer, and denied the use of any illicit or recreational substances. He had started walking back home since his house was close to the pub. At one point, he felt he could get home faster by climbing a fence, so he decided to climb the fence. As he climbed to cross over, his foot got caught on the barbed wire, and he was stuck and entrapped on the fence. He held the wall tightly with his forearms to avoid falling off and kept shouting for help. However, the police attended to him after about three hours.

His initial triage vital signs were a pulse rate of 115 bpm, blood pressure of 154/90 mmHg, and respiratory rate of 16 breaths per minute. His oxygen saturation was 97% on room air, and his temperature was 36.5°C. He did not have any past medical history. On examination, he was in a hospital wheelchair, conscious and alert, with a Glasgow Coma Scale score of 15 out of 15, and in distress from pain, unable to stand due to severe muscle pain (myalgia), swollen ankles, and painful knees (arthralgia), and the left knee was worse than the right. His lower leg was flexed at the knee, and he could not extend the knees. He had a positive straight leg raise in both limbs, unable to bear weight. His forearms were severely bruised and ecchymotic. There were no sensory deficits. His respiratory and cardiovascular examinations were normal.

The initial urine dipstick showed dark brown tea-colored urine with marked hematuria (+++) despite the urine microscopy not showing any red blood cells. Urine volume was about 1 liter at presentation. X-rays of his left ankle and knee were normal. His venous blood gas showed a pH of 7.38, bicarbonate of 17.5 mmol/l, base excess of -6.6, lactate of 3.58 mmol\l, and blood sugar of 4.5 mmol/l. His ECG showed normal sinus rhythm. Blood investigations were done, which showed CK of 41,654 IU/L, average cut-off value of 40-320 IU/L, abnormal liver function (raised alanine aminotransferase (ALT) of 183 U/L), and normal renal function (see Table 1). A diagnosis of rhabdomyolysis was made. A nasopharyngeal swab for SARS-CoV-2 was negative, and a polymerase chain reaction for the COVID-19 virus was also negative.

**Table 1:** Electrolytes, kidney function, inflammatory markers, and muscle enzyme progression throughout the hospitalization period. eGFR: estimated glomerular filtration rate.

Urea & electrolytes	Reference range (units)	Day 1	Day 2	Day 3	Day 4	Day 5	Day 6	Day 7	Day 8	Day 9
Sodium	133-146 mmol/L	137	135	139	137	139	139	134	136	138
Serum potassium	3.5-5.3 mmol/L	4.8	4.6	4.3	4.3	4.4	4	4	3.9	3.9
Urea	2.5-7.8 mmol/L	5.5	4.8	4.7	3.2	4.3	5.1	4.3	3.7	3.6
Creatinine	60-110 umol/L	79	81	82	77	83	79	71	85	75
eGFR/1.73 m2	Greater or equal to 60 ml/min	119	116	114	120	112	119	124	109	116
Serum creatine kinase	40-320 U/L	41654	79539	81917	74828	21192	11338	5003	1264	525
Plasma C-reactive proteins	0-5 mg/L	3	-	64	69	12	9	40	50	30

**Figure 1:**
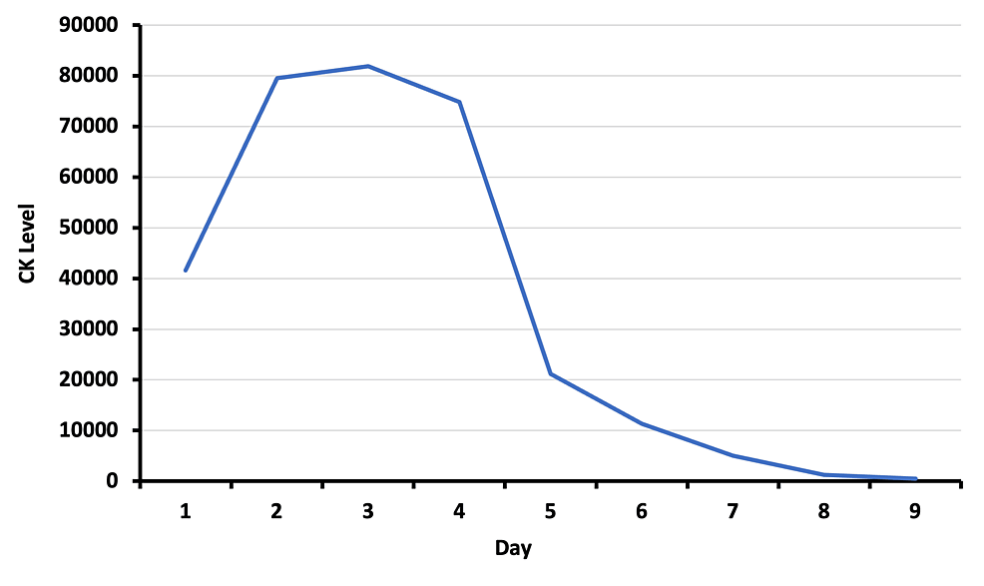
Change in creatine kinase (CK) level over the hospitalization period.

The patient was promptly admitted to the hospital and started on intensive intravenous fluid therapy to ensure hydration and to prevent acute kidney injury. Pain relief was initiated with two tablets of oral co-codamol 30/500 mg, which did not alleviate the pain. Thus, intravenous morphine 10 mg stat was started. He was also treated with alkalinization of urine to minimize myoglobin-induced renal damage. Close monitoring of renal function, electrolytes, and fluid balance was carried out throughout his hospital stay. When reviewed later in the morning, the patient could not stand or mobilize despite adequate analgesia. Repeat blood after six hours of initial investigation showed normal renal function with CK of 79,539 IU/L, which increased to a max peak of 81,917 before starting to decline, as seen in Figure 1.

He was referred to the orthopedic team for possible compartment syndrome. However, compartment syndrome was clinically excluded. The patient responded well to the treatment, and his symptoms gradually improved. He was discharged after 11 days of hospital admission, with instructions for close follow-up and further investigations to assess any long-term complications.

## Discussion

In the USA, the annual incidence of rhabdomyolysis is 25,000-38,000 cases [7], with a prevalence AKI of up to 30% [1]. This case illustrates that prolonged entrapment and immobilization, potentiated by non-traumatic heavy and binge drinking, can elaborate rhabdomyolysis [1,2]. Prolonged entrapment on a fence is an unusual cause of rhabdomyolysis. The mechanism of muscle injury in this case is likely multifactorial, involving direct mechanical trauma, immobilization, ischemia-reperfusion injury, and muscle compression.

Alcohol misuse is the most common cause of prolonged immobilization and coma in rhabdomyolysis [8]. Therefore, the direct toxic effects of alcohol in skeletal muscles through ethanol disruptive effect of ATP pump function, the disintegration of the muscle membrane, and alteration of the sarcoplasmic retinaculum or induction of the cytochrome P450 are involved in the elaborations of the rhabdomyolysis [9]. The degradation of creatine phosphokinase and its removal from the plasma is slow. Hence, the concentration of CK remained elevated longer and is consistent with the severity of muscle necrosis. In our patient's urine, myoglobin was positive, and serum CK peaked at 81,917 U/L by the third day despite fluid resuscitation. By the ninth day, his CK had dropped significantly to 525 IU/L.

Rhabdomyolysis is associated with hyperkalemia, hypocalcemia, hyperuricemia, and hyperphosphatemia and thus can lead to potentially life-threatening complications, such as AKI, electrolyte imbalances, cardiac arrhythmias, and disseminated intravascular coagulation [8,10]. The patient presented early with clinical and biochemical evidence of rhabdomyolysis. However, his acid-base statuses and electrolytes were surprisingly normal. His liver function tests rose from ALT of 183 U/L on the day of admission to 426 U/L and 457 U/L on days four and five of admission, respectively. However, the liver enzymes showed marked improvements after the 10th day when levels started to drop.

**Figure 2:**
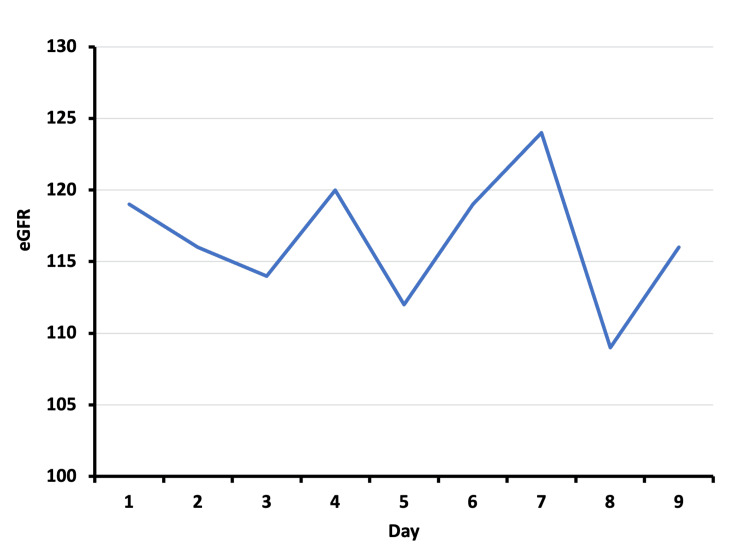
The change in eGFR over the hospitalization period. eGFR: estimated glomerular filtration rate.

He was commenced on fluid resuscitation, mainly saline 0.9%, starting with a rate of 400 mL/hour to preserve renal function while monitoring urine output, potassium, and CK levels, aiming at a urine output of 1-3 mL/kg/hour. As shown in Figure 2, by day four, the eGFR was optimum at 120 ml/min but showed a drop to 112 ml/min when fluid therapy had reduced. In furtherance, the renal function was reduced to 109 ml/min on the 7th day of admission. However, with aggressive fluid therapy, the renal function reverted to normal at 116 ml/min prior to being discharged home.

The patient also had optimization of analgesia while in the emergency department within the first three hours of muscle injury. Urine alkalinization (using 50 mEq of sodium bicarbonate added to 500 ml of 0.9% normal saline) prevents precipitation of myoglobin in the distal convoluted tubules of the kidney, decreases the precipitation of uric acid, corrects metabolic acidosis, and decreases the risk of hyperkalemia [11]. The aim is to maintain a serum and urine pH of 7.5 and 6.5, respectively. Early recognition, prompt management, and preventive measures are crucial in minimizing the risk of complications associated with this condition.

## Conclusions

Rhabdomyolysis can be a life-threatening multi-system condition with massive CK rise, electrolyte disturbances, AKI, cardiac arrhythmias, and disseminated intravascular coagulation. Patients who have experienced immobilization, crush injuries, alcohol consumption, or illicit drug use are clearly at risk of developing rhabdomyolysis. Therefore, it is evident that in our patient, a combination of physical exertion, muscle injury from entrapment, lack of mobility, and alcohol intoxication were the factors responsible for his rhabdomyolysis. Paralysis and severe weakness may suggest very extensive myonecrosis or coexistent potassium disturbances that can occur as AKI is impaired. The primary goal for treating rhabdomyolysis is fluid resuscitation to restore intravascular volume, dilute myoglobin and other toxins, restore kidney function, and prevent cardiac dysrhythmias. Finally, the patient was referred to the orthopedic team for possible MRI and surgical decompression by fasciectomy since the delay is likely to lead to irreversible damage. In summary, emergency physicians should be aware of the combination effects of alcohol-induced rhabdomyolysis and entrapment and immobilization causes of rhabdomyolysis, as early recognition and treatment can prevent the progression of life-threatening complications and reduce the need for renal dialysis.
